# Recent advances in dermoscopy

**DOI:** 10.12688/f1000research.7597.1

**Published:** 2016-02-17

**Authors:** Teresa Russo, Vincenzo Piccolo, Aimilios Lallas, Giuseppe Argenziano

**Affiliations:** 1 Dermatology Unit, Second University of Naples, Naples, Italy; 2 First Department of Dermatology, Aristotle University of Thessaloniki, Thessaloniki, Greece

**Keywords:** Dermoscopy, melanoma, basal cell carcinoma, squamous cell carcinoma, general dermatology

## Abstract

The use of dermoscopy has offered a new morphological dimension of skin lesions and has provided an effective diagnostic tool to differentiate melanoma from other benign or malignant skin tumors but also to support the clinical diagnosis in general dermatology. The aim of this article is to provide an overview of the most recent and important advances in the rising world of dermoscopy.

## Introduction

Dermoscopy is a non-invasive diagnostic procedure that allows a rapid *in vivo* evaluation of morphologic structures of the epidermis, the dermoepidermal junction, and the papillary dermis, not visible to the naked eye. Given that dermoscopic structures have been assessed to correlate well with the underlying histopathologic alterations, the method can be regarded as a link between clinical and histopathologic examination. Nowadays, the dermatoscope is considered the dermatologist’s stethoscope and its use has become very popular for both dermatologists and their patients, who often seek dermoscopic examination even when it is unnecessary ^1, 2^. Dermoscopy was first introduced to improve melanoma detection, and the evaluation of pigmented and non-pigmented skin tumors still represents its most important indication. However, the continually increasing descriptions of the dermoscopic patterns of several infectious and inflammatory skin diseases steadily establish an essential role for dermoscopy in all fields of dermatology ^2^.

The beneficial role of dermoscopy in improving melanoma diagnosis has been established at the highest possible level of evidence. However, even after dermoscopic examination, some melanomas might escape detection, either because of their morphologic characteristics or because of the overall patient’s context. In our estimation, the most diagnostically challenging scenarios are the following: melanoma in a patient with multiple moles, slow-growing melanoma (SGM), lentigo maligna (LM), nodular melanoma (NM), and amelanotic melanoma.

## Melanoma in patients with multiple moles and slow-growing melanoma

Recent evidence confirmed the widespread belief that clinical examination, coupled with dermoscopy, allows the recognition of the majority of melanomas. Specifically, approximately 80% of melanomas are easily recognized on the basis of their clinical or dermoscopic morphologic characteristics, or both. The remaining 20% of melanomas, in contrast, may be missed at the first consultation, since they lack dermoscopic characteristics allowing their discrimination from nevi. The latter is especially relevant in the context of patients with multiple clinically atypical moles, among which melanoma might be perfectly hidden. Effectively, the management of patients with the so-called “atypical mole syndrome” is highly challenging. Excising all (or many) atypical moles is absolutely meaningless, since this strategy induces significant morbidity without reducing at all the risk of melanoma. The optimal strategy for the management of such patients includes total body photography, digital dermoscopic documentation, and periodic monitoring. At the baseline visit, the detailed dermoscopic examination allows the identification of the so-called “signature pattern” of the patient’s nevi, which is based on the observation that the vast majority of an individual’s nevi display similar dermoscopic characteristics. The identification of the patient’s signature nevus pattern is extremely useful because it allows the correct classification of nevi as such while enabling the early recognition of melanoma, which usually deviates from the predominant dermoscopic pattern (ugly duckling or comparative approach). At follow-up visits, clinicians acquire information on the morphologic evolution of lesions with time, which allows the recognition of approximately 10% of melanomas that are morphologically featureless. To optimize the patients’ compliance, the first re-examination should be scheduled at 3 months after the baseline visit, and the following visits at 6- to 12-month intervals. In patients with multiple nevi, a regular annual follow-up is the only safe strategy to detect indolent SGMs characterized by subtle changes over time, which can be recognized only by a prolonged surveillance ^1, 2^. A recent study reported on the morphologic evolution of 92 featureless SGMs followed for at least 12 months prior to excision. They have noticed that most of them had minimal (i.e. not more than 2 mm) or no change in size during follow-up but became more disorganized, revealed loss of network in favor of structureless areas, developed a negative network, and exhibited new colors, including dark-brown, black, gray, blue, red, and white ^3^.

## Lentigo maligna

Clinical recognition of LM remains one of the most difficult tasks of clinicians, even with the addition of dermoscopy. This diagnostic trouble is related to the fact that LM, pigmented actinic keratosis (PAK), solar lentigo/seborrheic keratosis (SL/SK), and lichenoid keratosis (LPLK) often display overlapping dermoscopic criteria. Comparative studies between LM and SL/SK identified a set of four criteria predictive for LM diagnosis: asymmetric pigmented follicular openings, dark rhomboidal structures, slate-gray globules, and slate-gray dots. In contrast, SL/SK is dermoscopically typified by a sharp demarcation, moth-eaten borders, and fingerprinting. The discrimination between LM and PAK is much more problematic, since the latter has been shown to potentially exhibit all LM criteria. Effectively, a biopsy is often required to differentiate between LM and PAK. Similarly, histopathologic examination represents the only efficient method to classify pigmented facial lesions with extensive regression, where the differential diagnosis lies between regressed melanoma and LPLK (regressed SL/SK). This is because the dermoscopic regression structures of LM and SL/SK are identical structures (gray granules and white areas).

Three simple rules may help to minimize the risk of inappropriate diagnosis and management of LM: (1) the predominance of gray color in facial pigmented macules represents an important alarm sign because it reflects melanin deposition on the upper dermis and within the hair follicles; (2) biopsies of pigmented facial lesions should always be dermoscopy-guided, whereas clinical, dermoscopic, and histopathological findings should always be integrated (i.e. a histological result of a “junctional nevus” on the face of an elderly patient must be surely reviewed); and (3) ablative treatments (e.g. cryotherapy, laser therapy, and so on) should be avoided on equivocal facial lesions ^4^.

## Nodular melanoma

In contrast to SGM, NM is a rapidly progressing neoplasm that accounts for 10% to 30% of all melanomas and nearly 50% of all melanomas thicker than 2 mm. NM is characterized by a very aggressive biologic behavior, rapidly progressing (or even starting with) a vertical growth phase.

Unfortunately, NM is frequently not diagnosed until progressing to an advanced stage, resulting in a highly unfavorable prognosis. The difficulty in NM recognition results from the fact that it lacks the clinical ABCD criteria (asymmetry, border irregularity, color variegation, and diameter of more than 6 mm), often developing as a perfectly symmetric tumor in terms of shape and color. To address this problem, the “EFG” rule (elevation on cutaneous plane, firmness on palpation, and growth continuous over 1 month) has been introduced in clinical practice to enable the detection of this aggressive melanoma type.

The recognition of NM is also difficult dermoscopically, since the tumor often lacks the well-known melanoma-specific criteria, whereas available evidence on dermoscopy of NM is relatively scarce. However, during recent years, some studies attempted to test and validate dermoscopic criteria associated with this aggressive melanoma subtype ^5^. Argenziano *et al.* ^6^ introduced the “blue-back rule”, suggesting that the simultaneous presence of blue and black areas involving at least 10% of the lesion surface each were significantly associated with pigmented NM. Blue color is usually seen as structureless areas, corresponding to aggregations of pigmented melanocytes in the deep dermis. Black color may be seen as dots, globules, or blotches, which result either from superficial (intraepidermal) melanin or from dense dermal proliferations of pigmented melanocytes under a thinned (often because of ulceration) epidermis ^6^. This is in line with the observation that ulceration is more frequent in NM compared with superficial spreading melanoma.

Zalaudek *et al.* suggested that “atypical” vascular structures, including polymorphic vessels, milky red areas, and homogeneous red areas, are also significantly associated with NM ^7^.

In conclusion, although NM often lacks the “classic” melanoma-specific criteria, dermoscopy might enhance its recognition by revealing blue and black color or abnormal vascular structures or both.

## Amelanotic and hypomelanotic melanoma

Amelanotic and hypomelanotic melanoma are relatively rare, accounting for less than 2% of all melanomas. Their clinical recognition is particularly difficult, since they might mimic several benign hypopigmented skin lesions, often resulting in a significant delay in diagnosis and deterioration of prognosis. Amelanotic melanoma might develop as a reddish to pinkish macule, papule, plaque, or nodule that rapidly changes in size, shape, and color. Hypopigmented melanoma displays small foci of pigmentation, more frequently located at the periphery of the lesion. Their clinical differential diagnosis includes a variety of benign and malignant lesions ^8^, such as dermal nevus, pyogenic granuloma, adnexal tumor, Spitz nevus, vascular tumors, basal cell carcinoma (BCC), squamous cell carcinoma (SCC), keratoacanthoma, and Merkel cell carcinoma. Since the vast majority of melanoma-associated dermoscopic structures are pigmented, amelanotic melanoma is usually dermoscopically “featureless” and thus difficult to recognize. The most useful dermoscopic criteria are a milky red color and an atypical vascular pattern, consisting of either linear irregular vessels or dotted plus linear vascular structures. Especially in the context of nodular tumors, the only safe strategy not to miss amelanotic melanoma is to excise any lesion that cannot be safely diagnosed as benign after clinical and dermoscopic examination ^9, 10^.

## Basal cell carcinoma

The value of dermoscopy in improving the diagnosis of BCC has been extensively demonstrated over the last few decades. The most recent advances come from studies suggesting that dermoscopy significantly facilitates the accurate management of the tumor. Specifically, dermoscopy reveals tumor characteristics that might influence the treatment choice, such as the histopathologic subtype and the presence of ulceration or pigmentation.

In more detail, the dermoscopic criteria associated with non-pigmented BCC include arborizing vessels or fine telangiectasia with few ramifications, ulcerations or multiple small erosions, shiny white-red structureless areas, and short white steaks. The presence of fine telangiectasias with few ramifications or multiple small erosions (or both) predicts the superficial subtype, whereas the presence of arborizing vessels and large ulcerations predicts the nodular subtype.

Pigmented BCC is dermoscopically typified by multiple blue-gray dots/globules, in-focus dots, maple leaf-like areas, spoke wheel areas, concentric structures, and blue-gray ovoid nests. The dermoscopic detection of brown-colored structures, including maple leaf-like areas, spoke wheel areas, or concentric structures, is predictive of superficial BCC, whereas the presence of blue-gray ovoid nests predicts a non-superficial subtype.

Infiltrative BCC often exhibits white/red structureless areas, whereas the sclerodermiform BCC often displays a whitish background, corresponding to the underlying fibrosis.

As described above, dermoscopy provides useful and reliable information on the histopathologic BCC subtype, which is very important for tumor management ^11^. Specifically, when clinical and dermoscopic characteristics are suggestive of superficial BCC, the clinician could consider choosing a non-surgical treatment such as cryotherapy.

In addition to predicting the histopathologic subtype, dermoscopy might reveal morphologic characteristics of the tumor that are relevant for designing the treatment strategy. For example, the presence of multiple small erosions or ulcerations has been suggested to represent a predictor of favorable response to imiquimod. Another example is the potential of dermoscopy to reveal pigmentation in 30% of clinically non-pigmented BCCs, which is particularly relevant for BCCs scheduled to be treated with photodynamic therapy (PDT). This is because pigmented tumors are known to be less responsive to PDT, since melanin acts as a competitive light-absorbing pigment, thus reducing the tumor’s response rate.

When clinical and dermoscopic features of nodular, infiltrative, or sclerodermiform BCC are present, surgical excision undoubtedly represents the first choice to minimize the possibility of tumor recurrence. Moreover, dermoscopy, by providing a more accurate assessment of the true extension of the tumor, allows a more precise estimation of the required surgical margins, helping to minimize the recurrence rates ^11^. In conclusion, dermoscopy not only facilitates the clinical recognition of BCC but also provides additional clues to guide the correct management of the tumor.

## Keratinocyte skin cancers

The dermoscopic characteristics of the entities included in the spectrum of keratinocyte skin cancer have been recently investigated. Specific dermoscopic features have been suggested to characterize actinic keratosis (AK), intraepidermal carcinoma (Bowen’s disease), and invasive SCC.

A clinical classification of AKs into three grades has been recently introduced, and different dermoscopic criteria have been suggested to characterize each clinical grade: a red pseudonetwork typifies grade 1 AKs, a strawberry pattern is characteristic of grade 2, and structureless white to yellow areas and keratotic follicular openings are usually seen in grade 3 tumors. Pigmented AKs additionally display a superficial brown network surrounding the follicular openings ^12^.

Dermoscopy of non-pigmented intraepidermal carcinoma (Bowen’s disease) reveals glomerular vessels that are arranged in clusters and white to yellow scales. Pigmented Bowen’s disease may also display thick, brown dots with a linear arrangement, usually seen at the periphery of the lesion.

The dermoscopic pattern of invasive SCC has been shown to depend on the grade of histopathologic differentiation ^13^. In particular, well-differentiated SCC displays signs of keratinization as opaque, yellow scales, a central mass of keratin, structureless white areas, and yellow keratotic follicular plugs surrounded by a white rim (white circle). Linear irregular and, mainly, hairpin vessels might also be seen at the periphery of the tumor, especially in the keratoacanthoma type. In contrast to well- and moderately differentiated SCC, poorly differentiated subtypes commonly lack signs of keratinization, displaying a predominant red color, which results from dense vascularity. Pigmented SCC might reveal a homogeneous pigmentation, irregular blue-gray granular structures, or dark-brown to black crusts when ulcerated ^11^. In conclusion, dermoscopy is useful for diagnosing different stages of keratinocyte skin cancer, improving the optimal tumor management accordingly.

## Dermoscopy in general dermatology

Beyond the well-known value of dermoscopy for the diagnosis of skin tumors, its role in general dermatology is increasingly gaining appreciation among clinical practitioners ^14– 16^. The expansion of dermoscopy has been facilitated by the development of handheld polarized dermatoscopes, which are highly portable, do not require skin contact or immersion fluid, and allow fast screening of numerous lesions. Lately, several terms have been suggested to name the use of dermoscopy in different fields, such as trichoscopy for hair disorders, onychoscopy for nail abnormalities, entomodermoscopy for skin infestations, and inflammoscopy for inflammatory skin diseases. Among the several novel applications of dermoscopy, its utility for the diagnosis of inflammatory and infectious skin diseases attracts major interest among dermatologists, given the incidence of these disorders and the difficulties in differential diagnosis in everyday practice. Application of dermoscopy should follow the standard procedure of acquiring information from patient history and clinically evaluating the number, location, and morphology of the lesion(s). Four parameters should be assessed when applying dermoscopy in the realm of inflammatory and infectious diseases—(i) morphological vascular patterns, (ii) arrangement of vascular structures, (iii) colors, and (iv) follicular abnormalities—and the presence of other specific features (clues) should also be evaluated ^14– 16^. It must be underlined that dermoscopic findings should always be interpreted within the overall clinical context of the patient, integrated with information from the history and the macroscopic examination. Some dermoscopic criteria appear to be highly specific for a particular disease, whereas others can be seen in more than one entity and subsequently are considered “non-specific”. However, a “non-specific” feature may be rendered particularly valuable when coupled with certain other clinical dermoscopic criteria, forming a set of features that frequently leads to either an accurate single diagnosis or a narrowed list of possible differential diagnoses. Nowadays, common dermatologic diseases can be diagnosed by dermoscopy, which becomes particularly useful in cases of atypical or unusual manifestations. The best-studied disease is psoriasis, which dermoscopically always displays dotted vessels with regular distribution and white scales ^14– 16^. Dermoscopy makes differential diagnosis among papulosquamous disorders simpler, permitting clinicians to recognize lichen planus (Wickham striae), eczema (yellow crusts and patchy dotted vessels), and pityriasis rosea (peripheral white scales). Moreover, the detection of a yellow-orange background is considered a dermoscopic clue for the diagnosis of granulomatous skin disorders, such as sarcoidosis, lupus vulgaris, and necrobiosis lipoidica. Dermoscopy has been found useful in the diagnosis of rosacea, discoid erythematous lupus, morphea, lichen sclerosus, pigmented purpuric diseases, Darier’s disease, Grover’s disease, and porokeratosis. In regard to infectious diseases, both frequent (warts, molluscum contagiosum, mycosis, and mite infestations) and uncommon (myiasis and tinea nigra) diseases can be diagnosed by application of dermoscopy, and scabies represents the most striking example (delta-wing jet with contrail sign). Recently, practical tips have been suggested to enhance and optimize the use of dermoscopy by clinicians in their everyday practice of general dermatology. However, given that only a few appropriately designed studies have assessed the diagnostic accuracy of dermoscopy in fields other than skin tumors, these suggestions are based on the expert opinions of a group of clinical and research dermoscopists and should be read with a critical eye, pending further higher-level evidence.

## Conclusions

In the early years of dermoscopy, the method was considered a second-level tool for further evaluating suspicious skin tumors. With several data and considerable experience gathered in recent decades, the role of dermoscopy became totally different. Today, the dermatoscope is an irreplaceable clinical tool used for the evaluation of virtually every skin lesion, completing the puzzle of clinical examination. Furthermore, the importance of dermoscopy, one of the most popular and dynamic fields of research, in everyday clinical practice is expected to continually increase in the coming years.
